# Hard limits to cognitive flexibility: ants can learn to ignore but not avoid pheromone trails

**DOI:** 10.1242/jeb.242454

**Published:** 2021-06-04

**Authors:** Katharina Wenig, Richard Bach, Tomer J. Czaczkes

**Affiliations:** 1 Department of Behavioural and Cognitive Biology, University of Vienna, 1090 Vienna, Austria; 2 Animal Comparative Economics Laboratory, Department of Zoology and Evolutionary Biology, University of Regensburg, 93053 Regensburg, Germany

**Keywords:** Anti-instinctive learning, Associative learning, Chemical communication, Opposite-instinctive learning, Social signal

## Abstract

Learning allows animals to respond to changes in their environment within their lifespan. However, many responses to the environment are innate, and need not be learned. Depending on the level of cognitive flexibility an animal shows, such responses can either be modified by learning or not. Many ants deposit pheromone trails to resources, and innately follow such trails. Here, we investigated cognitive flexibility in the ant *Lasius niger* by asking whether ants can overcome their innate tendency and learn to avoid conspecific pheromone trails when these predict a negative stimulus. Ants were allowed to repeatedly visit a Y-maze, one arm of which was marked with a strong but realistic pheromone trail and led to a punishment (electric shock and/or quinine solution), and the other arm of which was unmarked and led to a 1 mol l^−1^ sucrose reward. After ca. 10 trials, ants stopped relying on the pheromone trail, but even after 25 exposures they failed to improve beyond chance levels. However, the ants did not choose randomly: rather, most ants began to favour just one side of the Y-maze, a strategy which resulted in more efficient food retrieval over time, when compared with the first visits. Even when trained in a go/no-go paradigm which precludes side bias development, ants failed to learn to avoid a pheromone trail. These results show rapid learning flexibility towards an innate social signal, but also demonstrate a rarely seen hard limit to this flexibility.

## Abstract (precis)

**Summary:** Although *Lasius niger* ants immediately learn to avoid odours, they fail to avoid pheromone trails when they are associated with a punishment; however, they learn to ignore trails by developing simple heuristics.

## INTRODUCTION

Organisms can respond to predictable changes in the world in different ways, and over many time scales. Evolution can shape hard-wired adaptations, including behaviours, in response to environmental change occurring over many generations. Pleiotropic effects can allow animals to respond to changes in the world more rapidly, between single generations. However, these adaptations cannot help organisms cope with predictable events that change within their own lifetime. Learning offers such flexibility (Shettleworth, 2009). However, learning comes at a cost: it requires not only neural architecture to be grown and maintained but also a period of information collection, before which it is ineffective (Dukas, 1999; Dunlap and Stephens, 2016). This leaves animals exposed to making poor decisions until learning has taken place. Thus, learning is not predicted to occur for responses that are always appropriate. Studying where learning does or does not occur can thus inform us about the evolutionary pressures on an organism.

In addition to learned responses, animals are equipped with a range of innate responses, which do not require learning. However, although these do not require learning, they are often open to it – these responses can be modified. For example, flies innately explore novel environments incessantly, but if repeatedly punished for moving, reduce their movement (Sun et al., 2020; ‘operant conditioning’: Skinner, 1963). Similarly, crayfish express an innate positive taxis towards blue light but learned to avoid it for at least 48 h after three pairings with electric shocks (Okada et al., 2021).

Pheromones are chemical signalling substances, triggering innate responses (Karlson and Lüscher, 1959; Sudd, 1959; Wyatt, 2014; but see Baracchi et al., 2020). However, studies on pheromone-mediated reproductive behaviours (for reviews, see Beny and Kimchi, 2014; Pfaus et al., 2001) have demonstrated the role of past experiences and learning in the modification of those responses. For example, male mice reduced the production of ultrasonic vocalizations in response to female urine presentation when urine presentation was repeatedly not followed by the female herself (Nyby et al., 1978). Male golden hamsters were shown to suppress pheromone-mediated sexual behaviours towards females after vaginal secretion presentation was followed by lithium chloride poisoning (Johnston and Zahorik, 1975; Zahorik and Johnston, 1976). However, not only does it seem possible to modify the frequency of innate behaviours after punishment via operant conditioning but also animals can learn to act against their innate response by changing the representation of the valence associated with certain stimuli (‘anti-instinctive learning’): rats innately react aversively towards peppermint odour and cadaverine but when associating the odours with a positive stimulus (e.g. a tactile stimulation that mimics suckling in pups: Yuan et al., 2013; or sexual behaviour in adult males: Pfaus et al., 2012), the valence of the odours can positively shift and even become attractive (‘classical conditioning’: Pavlov, 1927). Honeybees, as another example, are innately attracted to geraniol and citral, two major components of the Nasanov pheromone (Pickett et al., 1980). However, when these substances are paired with electric shocks, bees eventually begin to show aversive responses (sting extension) when exposed to these pheromones alone and efficiently retrieve the learned association 1 h after the initial test (Roussel et al., 2012). The last example is one of the very few demonstrations of anti-instinctive learning in an insect. However, while the valence response was reversed (something positive became negative), the behavioural response modality was different: a shift from a locomotion response (attraction) to a defensive response (sting extension). To date, only very few examples of anti-instinctive learning in the same behavioural response modality have been demonstrated in mammals (Pfaus et al., 2012; Yuan et al., 2013), and it is unclear whether insects would be capable of such a complete behavioural reversal (opposite-instinctive learning).

Many ants, as well as termites, bees and wasps, deploy pheromone trails to guide nestmates to important resources (Bordereau and Pasteels, 2010; Czaczkes et al., 2015; Jeanne, 1981; Lindauer and Kerr, 1958). Trail following is overwhelmingly considered an innate behaviour which does not require learning (but see Cammaerts, 2013; Reichle et al., 2011). However, the response to pheromone trails is not always full and absolute: other information sources affect how organisms respond to such trails (Czaczkes et al., 2015). For example, ants may ignore pheromone trails if they conflict with memory (Cronin, 2013; Grüter et al., 2011; Harrison et al., 1989), or use orientation cues to decide in which direction to follow a trail if they join it in the middle (Czaczkes and Ratnieks, 2012; Minoura et al., 2016). Ants are also good learners, and well-able to rapidly form associations between odours and rewards or punishments (Desmedt et al., 2017; Dupuy et al., 2006; Oberhauser and Czaczkes, 2018; Turner, 1907). In order to examine opposite-instinctive learning in *Lasius niger* (henceforth ‘ants’), four experiments were conducted. First, we attempted to teach ants that a pheromone trail predicted a quinine punishment on one arm of a Y-maze while the unmarked arm led to a sucrose reward (experiment 1). As ants in experiment 1 quickly learned to avoid punishment by carefully probing the quinine drop with their antennae without tasting it, we developed an apparatus for delivering inescapable electric shock punishments to free-walking ants and used this in addition to quinine in experiment 2. Then, to exclude the possibility that side-bias learning was preventing opposite-instinctive learning, we examined whether ants could learn that the pheromone predicted a punishment using a go/no-go paradigm on a linear runway (experiment 3). Finally, we demonstrated that ants can learn to avoid an odour when it predicts a negative stimulus (experiment 4).

## MATERIALS AND METHODS

### Study species and maintenance

We used 16 queenless colony fragments (henceforth ‘colonies’) of the black garden ant, *Lasius niger* (Linnaeus 1758) collected from 16 different mother colonies on the University of Regensburg campus. *Lasius* *niger* derive much of their carbohydrate intake from tending honeydew-producing insects (Eidmann, 1927) but do not show task specialization within aphid tenders (e.g. to guards, shepherds and transporters; Novgorodova, 2015). The colonies were housed in 40×30×20 cm plastic boxes with a layer of plaster covering the bottom. Each box contained a circular plaster nest (14 cm diameter, 2 cm high). The colonies contained around 1000–2000 workers and small amounts of brood. Queenless colonies forage and lay pheromone trails, and are frequently used in foraging experiments (Detrain et al., 2019). As foragers rarely interact with the queen (Stroeymeyt et al., 2018), the lack of queen (but not brood; see Portha et al., 2004) should have little effect on the details of forager behaviour. The colonies were fed *ad libitum* on 0.5 mol l^−1^ sucrose solution and received *Drosophila melanogaster* fruit flies once a week. Colonies were deprived of food 4 days prior to the experiments in order to achieve a uniform and high motivation for foraging and pheromone deposition (Josens and Roces, 2000; Mailleux et al., 2006). Water was always available *ad libitum*.

### Experimental setup and procedures

#### Experiment 1: learning to avoid pheromone trails – quinine punishment

##### Setup

**Fig. 1.**
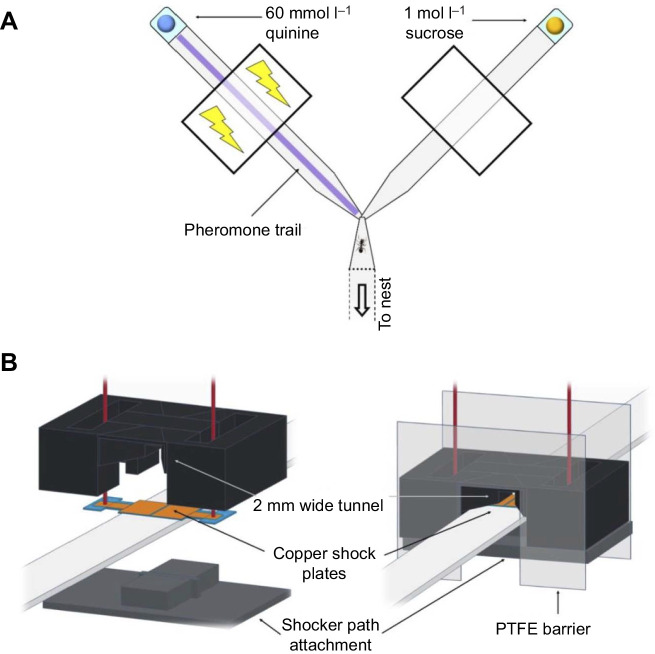
**Experimental setup.** (A) The Y-maze used in experiments 1, 2 and 4 and in the unrewarded learning tests in experiment 3. The arms were 10 cm long and 1 cm wide. The ant is shown to scale. The specific setup shown is experiment 2, where ants were punished on the pheromone-marked arm using both an electric shock (see B) and quinine solution. Experiment 1 was the same as experiment 2, but without the electric shock device (‘shocker’). In experiment 4, the unscented arm was rewarded, and instead of pheromone the punished arm was scented with lemon. The sides of the positive and negative arms (left/right) were pseudo-randomized between trials. (B) Schematic diagram of the shocker. An attachment part was affixed to the bottom of the path, and the shocker attached via magnets to allow rapid removal for cleaning and paper overlay replacement. The shocker was 3D printed from PLA (see Materials and Methods).

Experiment 1 explored whether ants could learn to avoid pheromone trails after the trails were associated with punishment. We tested 31 ants using a Y-maze (Fig. 1A) and marked its negative arm with a pheromone trail solution, by drawing 6 µl of the solution in an even line over the overlay using a calibrated capillary tube. This produces a strong but realistic pheromone trial, and elicits trail following indistinguishable from natural trail following (von Thienen et al., 2014). The positive arm was covered with a dichloromethane trail (6 µl) as a solvent control. A droplet of bitter tasting quinine solution (60 mmol l^−1^; Avarguès-Weber et al., 2010) was placed at the end of the negative arm and a droplet of 1 mol l^−1^ sucrose solution at the end of the positive arm of the Y-maze.

The pheromone solution was created by freeze-killing workers and dissecting out their hindguts – the glandular source of the *L. niger* pheromone trail (Bestmann et al., 1992; von Thienen et al., 2014). Four glands were macerated in 2 ml dichloromethane. The pheromone solution was separated into 1 ml aliquots and stored at −20°C between experiments. During the experiment, the aliquot used was kept on ice. Dichloromethane and pheromone trails were applied to the paper layers just before placing them on the Y-maze arms. Between trials, all paper layers were replaced to remove any pheromones left by the focal ant during the previous trial.

##### Procedure

To start the experiment, we touched a small piece of paper to the nest floor. The first ant to climb onto the paper was put on the starting point of the Y-maze, which had the positive arm covered with the control solvent, and the negative arm covered with a pheromone trail. After reaching the sucrose drop on the positive arm, the ant was marked with acrylic paint on its abdomen while drinking the sucrose solution. Afterwards, she was allowed back to the nest. After unloading the sugar, the ant was brought back onto the Y-maze for the next trial. In each of the following 25 trials, the ant's initial choice (left or right) was recorded when the ant crossed a line 1 cm from the bifurcation, while the final choice was recorded when the ant reached one of the droplets (quinine on the negative arm, sucrose solution on the positive arm). As ants ran on the runway, we also measured the time the ants took from entering the runway to reaching the sucrose solution on the positive arm.

The positive and negative arms and their respective reinforcement and punishment were switched between trials, following a pseudo-randomized order [L–R–L–R–R–L–L–R–R–R–L–R–L–L–R–R–L–L–L–R–R–L–L–R–R and its reversed sequence, where L (left) and R (right) indicate the arm of the maze containing the sucrose reward] to ensure the subjects would not associate the reward and punishment with any particular side.

#### Experiment 2: learning to avoid pheromone trails – quinine+shock punishment

##### Setup

During experiment 1, we noticed that ants very rapidly learned to carefully probe the droplet with their antennae before drinking. This enabled them to avoid punishment for an incorrect choice, thus greatly reducing the cost of errors. Such reduced error costs may not have been sufficient to promote learning. We thus designed a further experiment (experiment 2) in which we introduced an electric shock device (henceforth ‘shocker’; Fig. 1B) as an unavoidable punishment. Shockers were affixed 4 cm from the bifurcation on each of the Y-arms. They consisted of a 3D-printed PLA body (an STL file can be downloaded in modifiable form from https://www.tinkercad.com/things/dl5ce7taHaI-ant-zapper), which offered a tunnel narrowing from 1 cm (the width of the Y-maze arms) to a 2 mm gap (Fig. 1B). The floor of the gap was covered with two slightly disconnected copper plates (Fig. 1B, left), which were connected via wires to a button and a laboratory power supply. When the ant walked through the gap, thereby touching each copper plate with at least one of her legs, she closed the electric circuit (if the shocker was activated) and got shocked with 7.5 V (Roussel et al., 2012). The front and back of the shocker were equipped with a polytetrafluoroethylene (Fluon^®^) plastic plate, preventing ants from passing the barrier except via the tunnel. Apart from adding the shocker, experiment 2 was identical in design to experiment 1 above. A total of 31 ants were tested.

##### Procedure

While the overall procedure of experiment 2 was identical to that of experiment 1 above, we added two methodological improvements. Firstly, we established two pre-training trials prior to the trials, ensuring a standard baseline experience across subjects. Secondly, we added two unrewarded learning tests after trial 20 and 25, to increase the chance of uncovering any cryptic learning that may have occurred (Bortot et al., 2019).

In the pre-training trials, we confronted the ant with one rewarded trial followed by one punished trial, both presented on a linear runway (21 cm long, 1 cm wide). In the first trial, paper overlays covered with a control solvent were placed on the runway, which was also equipped with an inactive shocker and a droplet of sucrose solution that was presented behind the device. After reaching the sucrose drop, the ant was marked with acrylic paint on its abdomen while drinking the sucrose solution. Afterwards, she was allowed back to the nest. After unloading the sugar, the ant was allowed back onto the linear runway for a second pre-training trial. In this second trial, the ant was confronted with a linear runway covered with a pheromone trail, an activated shocker, and a droplet of quinine solution behind the device. After the ant experienced both negative stimuli (shock and quinine), it was transferred to the Y-maze for the test. Within the pre-training trials, the punishment trial was always carried out after the reinforcement trial to ensure the ant's participation.

During unrewarded learning tests, the arms of the Y-maze were equipped with the respective paper layers (control solvent or pheromone trail), but both shockers were inactivated, and the sucrose and quinine droplets were replaced by neutral water droplets. After entering one arm of the Y-maze, the connection to the Y-maze stem was interrupted for 1 min, therefore only allowing the exploration of both Y-maze arms. We recorded the overall time the subject spent on the correct (positive) arm before replacing the water droplets with the respective liquids (sucrose or quinine solution), allowing the ant to drink from the sucrose, and to return freely to the nest for further testing.

#### Experiment 3: learning to avoid pheromone trails – a go/no-go-paradigm

##### Setup

Ants in experiments 1 and 2 developed a strong but mostly arbitrary side bias (see Results). We were concerned that the ability to develop a favoured side may interfere with the task of learning to avoid the pheromone trail. We thus developed experiment 3, which excluded this possibility by employing a go/no-go paradigm on a linear runway. Twenty-nine ants were tested, using a 21 cm linear runway that was either marked with a pheromone trail (12 μl) and offered a droplet of bitter tasting quinine solution at the end (punishment trial) or marked with a dichloromethane trail (12 µl) and contained a droplet of 1 mol l^−1^ sucrose solution at the end (reward trial). As ants ran on the runway, we measured the time the ants took from entering the runway to reaching the droplet at the end as well as the number of U-turns they performed on their way to the droplet. A U-turn was defined as turning around and moving at least 1 cm towards the nest and away from the droplet. In addition, we carried out two unrewarded learning tests on a Y-maze, as described in experiment 2, and recorded the ant's choices (left or right). All trials were video-recorded, and videos were subsequently analysed by a naive coder, to ensure blindness to trial type (punishment or reward trial).

##### Procedure

To start the experiment, we touched a small piece of paper to the nest floor. The first ant that climbed onto the paper was put on the starting point of the linear runway. The first trial was always a reward, no-pheromone trial to ensure the ant's participation. A total of 25 trials were presented in a pseudo-randomized order (R–P–R–P–P–R–R–P–P–P–R–P–R–R–P–P–R–R–R–P–P–R–R–P–P, where R and P are the reward and punishment, respectively). When reaching the sucrose solution in the first reinforcement trial, the ant was marked with acrylic paint on its abdomen while drinking. Afterwards, the marked ant was brought back to the nest, where she could unload her sucrose load, and was then brought back to the start of the runway for a punishment trial with pheromone. After finding and tasting the quinine solution in the punishment trials, the ant was delayed for 30 s (the time it approximately took her to drink the sucrose solution in the reward trials), before she was brought back to the nest. After trial 10 and 20, unrewarded learning tests on a Y-maze were added, as in experiment 2.

#### Experiment 4: learning to avoid an odour

##### Setup

The aim of experiment 4 was to show that it is within the ants’ capacity to learn to avoid a neutral chemical signal (here: lemon odour) after it was associated with a punishment. We tested 12 ants using the same setup as in experiment 2 (Fig. 1) but replaced the pheromone trail on the negative arm of the Y-maze by lemon scent while the positive arm was covered with unscented paper overlays. Lemon-scented paper overlays were produced by placing unscented paper layers in an airtight container with lemon essential oil for at least 24 h; these were taken out of the container just before applying them to the runway. Between trials, all paper overlays (scented and unscented) were replaced to avoid orientation via pheromones left during the previous trial.

##### Procedure

Five ants were allowed to walk up a bridge, leading to a Y-maze, one arm of which was covered with an unscented paper overlay, and the other covered with lemon-scented paper. The first ant choosing the lemon-scented arm first was selected to be the subject, while ants choosing the unscented arm of the Y-maze were gently brought back into the nest. This was done in order to ensure that no ants with an innate aversion to lemon odour were tested, thus making the experiment very conservative. The pre-selected subject then carried out two pre-training trials, 12 trials [following a pseudo-randomized order: L–R–L–R–R–L–L–R–L–R–L–R and its reversed sequence, where L (left) and R (right) indicate the arm of the maze containing the sucrose reward] and two unrewarded learning tests after trial 8 and 12, all as described in experiment 2, but with lemon scent instead of a pheromone trail.

### Statistical analysis

The complete statistical analysis code for experiments 1–4 is available from figshare (https://doi.org/10.6084/m9.figshare.14141666.v1), as is the complete dataset used in the analysis (https://doi.org/10.6084/m9.figshare.14141669.v1).

We addressed the ants’ learning performance in experiments 1, 2 and 4 by running generalized linear mixed models (GLMMs) with binomial error distributions (McCullagh and Nelder, 1983), using the glmer function of the R package lme4 (Bates et al., 2015) in R version 4.0.2 (http://www.R-project.org/). We tested for the main effect of trial number (1–25 in experiments 1 and 2, 1–12 in experiment 4, 1 versus 2 in the unrewarded learning tests of experiment 3) on the proportion of final decisions of which route to take on the Y-maze (correct versus incorrect decisions) to investigate whether ants chose correctly more often with more experience.

Because the ant's initial decision (1 cm from the Y-maze bifurcation) and final decision (when reaching the reward or punishment) differed in only a very small proportion of trials (∼0.2% in experiments 1 and 2; ∼3.5% in experiment 4), we decided to only analyse the subjects’ final decisions. Initially, we used ant-ID, nested inside colony-ID, as a random effect in our models. However, because of convergence failure in these complex models, we decided to only include ant-ID as a random factor. The model formula was: Correct decision=trial number+(random slope: trial number, random effect: antID).

To evaluate potential side bias in experiments 1, 2 and 4, we ran GLMMs with binomial error distributions with trial number as a fixed effect, trial number as a random slope and ant-ID as a random effect, this time using the proportions of repeated decisions as the response variable. The model formula was: Repeated decision=trial number+(random slope: trial number, random effect: antID).

Linear models were used to assess the effect of (scaled) trial number on the (log-transformed) latency to reach the sucrose reward in experiments 1 and 2: (log) latency to sucrose=(scaled) trial number. Unrewarded learning tests in experiments 2 and 4 were evaluated using GLMM with binomial error distribution, testing for the main effect of trial number (1 versus 2) on the proportion of time spent on the correct (unscented) arm of the Y-maze. The model formula was: proportion of time spent on correct arm=trial number+(random slope: trial number, random effect: antID).

To analyse the learning performance of ants in experiment 3, we ran a linear mixed-effects model (LMM) with a Gaussian error distribution, including trial number (scaled; 1–25) and trial type (reward versus punishment trial) as main effects as well as their interaction, predicting the time (log-transformed) ants took to reach the respective droplet (quinine in punishment trials; sucrose in reward trials) at the end of the linear runway. We conducted a full *a priori* model examination (Forstmeier and Schielzeth, 2011) to check the overall effect of our test predictors and to avoid cryptic multiple testing. Trial number was added as a random slope and ant-ID as a random effect in our model for experiment 3. As U-turns occurred so rarely (∼6.4% of trials), we refrained from statistical analysis of this rather uninformative data. The model formula was: (log)latency to drop=condition_reward vs punishment_×(scaled) trial number+[random slope: (scaled) trial number, random effect: antID].

All statistical models were validated by examining the distribution of scaled residuals with the simulateResiduals function and testing for over- or under-dispersion using the ‘DHARMa’ package (https://CRAN.R-project.org/package=DHARMa). The alpha level for all analyses was set at *P*<0.05.

## RESULTS

The complete statistical analysis output for experiments 1–4 is available from figshare (https://doi.org/10.6084/m9.figshare.14141666.v1).

### Experiments 1 and 2: learning to avoid pheromone trails – quinine punishment and quinine+shock punishment

Experiments 1 and 2 were designed to test whether ants could learn to avoid a pheromone trail when it is associated with negative stimuli (experiment 1, avoidable punishment: quinine; experiment 2, unavoidable punishment: quinine and shock). The final dataset of experiment 1 was composed of 640 individual trials (ranging between 6 and 25 trials per ant), carried out by 31 ants from 16 different colonies; experiment 2 combined 603 individual trials (ranging between 3 and 25 trials per ant) by 31 ants from nine different colonies.

**Fig. 2.**
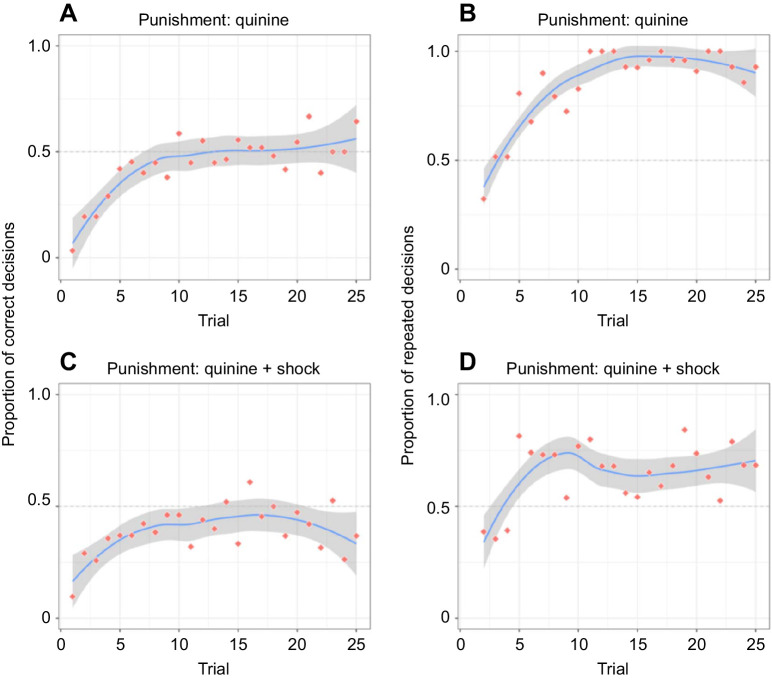
**Learning performance in the experiments.** (A,C) Learning performance (proportion of correct final decisions across 25 trials) in experiment 1 (avoidable punishment: quinine; A) and experiment 2 (unavoidable punishment: quinine+shock; C). (B,D) Formation of side bias (proportion of repeated final decisions across 25 trials) in experiment 1 (B) and experiment 2 (D). Red dots are individual trial means; blue lines are LOESS-smoothed means for the full 25 trial protocol; grey ribbons are bootstrapped 95% confidence intervals for the protocol mean lines. A+B: *n*=640 individual trials, 14–31 ants per trial; C+D: *n*=603 individual trials, 19–31 ants per trial.

**Fig. 3.**
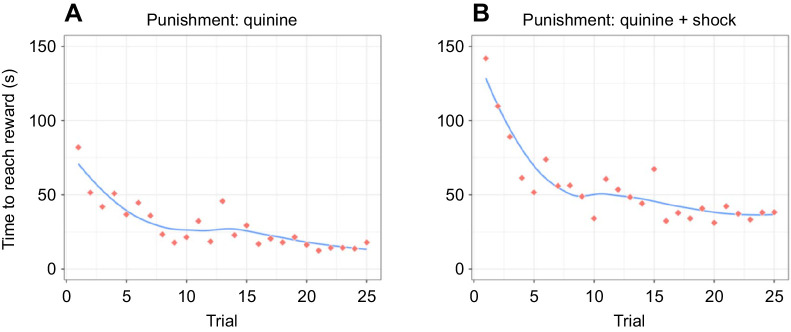
**Mean running time to reach the sucrose reward.** Data were obtained over 25 trials in experiment 1 (A) and experiment 2 (B), using a Y-maze. In experiment 2, it was not possible to distinguish between the effect of increased proficiency in the learning task and the effect of increased proficiency in navigating through the shocker. Red dots and blue lines as in Fig. 2. A: *n*=640 individual trials, 14–31 ants per trial; B: *n*=603 individual trials, 19–31 ants per trial.

When examining the ants’ choice accuracy (‘performance’ in figshare: https://doi.org/10.6084/m9.figshare.14141666.v1) over subsequent trials graphically, it became apparent that an increase in learning performance appeared only during the first (ca. 10) trials and then stabilized at around chance level (Fig. 2A,C). We thus split the dataset and ran two *post hoc* models, one for the first 10 trials and another for the last 15 trials. As expected from visual inspection of the data, ants’ performance increased over subsequent trials in the first 10 trials (experiment 1: *z*_trial_=4.87, *P*_trial_<0.001; experiment 2: *z*_trial_=3.30, *P*_trial_<0.001), but not in the subsequent 15 trials (experiment 1: *z*_trial_=0.69, *P*_trial_=0.489; experiment 2: *z*_trial_=−0.45, *P*_trial_=0.655). Importantly, while the choice accuracy of ants was lower than chance in the first 10 trials (experiment 1: *z*_intercept_=−6.41, *P*_intercept_<0.001; experiment 2: *z*_intercept_=−5.00, *P*_intercept_<0.001), choices in trials 11–25 were indistinguishable from 50% correct (experiment 1: *z*_intercept_=−0.62, *P*_intercept_=0.534; experiment 2: *z*_intercept_=−0.23, *P*_intercept_=0.818). However, ants significantly decreased their latency to reach the sucrose reward on the positive arm of the Y-maze (‘running times’ in figshare: https://doi.org/10.6084/m9.figshare.14141666.v1; *P*<0.001) across trials in both experiment 1 (Fig. 3A) and experiment 2 (Fig. 3B). Nonetheless, reduced latency times could be a result of increased proficiency in probing the drop in experiment 1, navigating through the tunnel of the shocker (see Fig. 1B) in experiment 2, and/or via increased familiarity with the overall setup in both experiments.

To further explore the ants’ behaviour, we examined their tendency to choose the arm they chose on the previous trial. Ants overwhelmingly chose the arm they had chosen on their previous trial (Fig. 2B,D), with this pattern rapidly developing over subsequent visits (‘side bias’ in figshare: https://doi.org/10.6084/m9.figshare.14141666.v1; experiment 1: *z*_trial_=6.49, *P*_trial_<0.001; experiment 2: *z*_trial_=2.51, *P*_trial_<0.05).

During the two unrewarded learning tests in experiment 2 (first trial after visit 20 and second trial after visit 25), in which subjects could freely explore both arms of the Y-maze for 60 s without receiving either reward or punishment, ants did significantly improve between trials (trial 1: 47.03%, trial 2: 77.77%, *z*_trial_=2.80, *P*_trial_<0.01; *z*_intercept_=−2.73, *P*_intercept_<0.01).

### Experiment 3: learning to avoid pheromone trails – a go/no-go-paradigm

**Fig. 4.**
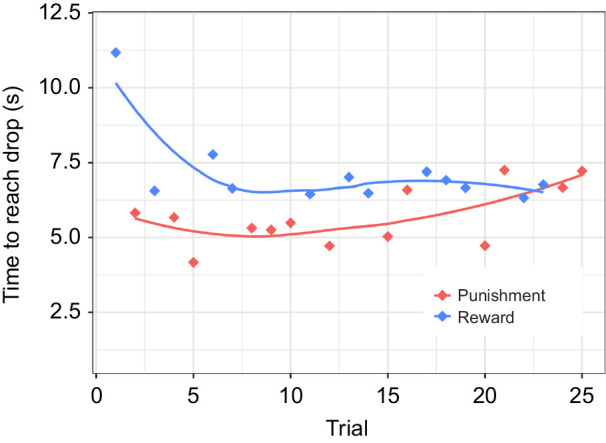
**Mean running time to the respective droplet in punishment and reward trials.** Data were obtained over 25 trials in experiment 3, using a linear runway. *n*=692 individual trials, 25–29 ants per trial.

Experiment 3 was designed to test whether ants could learn to avoid pheromone trails when developing a side bias was impossible. The final dataset was composed of 692 individual trials (ranging between 13 and 25 trials per ant), carried out by 29 ants from seven different colonies. The respective times to reach the drop changed significantly over consecutive trials in the punishment condition (*F*=4.37, *r*^2^=0.012, d.f.=358, *P*<0.05) but not in the reward condition (*P*_reward_=0.313). When assessing the interaction trial number×trial type (reward versus punishment), ants took increasingly longer to approach the presented quinine drop in punishment trials (∼1.19 s per trial; *P*<0.001; with reward trials serving as reference), showing some degree of learning. However, subjects overall remained faster in the punishment trials (Fig. 4), indicating hard limits to their learning performance.

In both unrewarded learning tests on a Y-maze, ants showed a significant preference for the pheromone-marked arm (*z*_intercept_=−3.25, *P*_intercept_<0.01) over the unmarked arm. However, the effect was significantly less pronounced in the second unrewarded learning test in comparison to the first unrewarded learning test (*z*_trial_=2.76, *P*_trial_<0.01; graph available from figshare: https://doi.org/10.6084/m9.figshare.14141666.v1).

### Experiment 4: learning to avoid odours

Experiment 4 was designed to test whether ants could learn to avoid a specific odour (here: lemon) after it was associated with a punishment. The final dataset of experiment 4 comprised 144 individual trials, carried out by 12 (for lemon preference pre-selected) ants from six different colonies. All 12 ants learned to avoid the lemon odour in the course of the initial pre-training trials (one reinforcement trial and one punishment trial) as all their final decisions during the following 12 trials were 100% correct. Furthermore, all ants spent 60 out of 60 s on the correct (unscented) arm of the Y-maze during the first unrewarded learning test (after trial 8); while two out of 12 ants briefly explored the lemon scented arm of the Y-maze during the second unrewarded learning test (after trial 12), lowering the mean time spent on the correct arm to 58.6 s (=97.6% of time spent on the correct arm)*.*

## DISCUSSION

Ants were adept learners, quickly learning to ignore pheromone trails (Fig. 2). However, we discovered a hard limit to their learning – while they could learn to ignore trails, they could not learn to actively avoid them. After 5–10 visits of rapid performance improvement, improvements stopped completely, and ants never increased choice accuracy above chance level. However, ants learned to probe the drop before attempting to drink it, and thus avoided the quinine punishment, which potentially restricted their learning success. We therefore conducted an experiment adding an unavoidable punishment by introducing a shocker (using a relatively high voltage, comparable to that used for harnessed honeybees, e.g. Roussel et al., 2009) but still ants did not improve above chance level in the given learning task. We can rule out a general inability of ants to learn to avoid chemical signals, as ants demonstrated one-trial learning with a consequent 100% accuracy in a comparable setup when the predictor for punishment was a lemon odour instead of a pheromone trail. These results indicate that odours could easily acquire negative valence through associative learning, while with pheromones, ants were unable to reliably perform opposite-valence responses.

However, ants did not choose randomly when confronted with the Y-maze: within the first 10 trials, most subjects started applying an alternative strategy and developed a side bias. This allowed them to improve their choice accuracy from ∼6.5% (initial choice accuracy) to ∼50% and shortened the latency to reach the sucrose reward (Fig. 3) over successive trials.

The display of repetitive behaviours and simple navigation rules, such as the formation of a side bias, has previously been described when ants are confronted with complex tasks (Macquart et al., 2008; Oberhauser et al., 2020). However, the ability to form side bias might have blocked the ants’ ability to learn to avoid pheromone trails in the presented task. We therefore confronted subjects with a go/no-go setup where the formation of a side bias was impossible, but ants still showed hard limits to their learning flexibility: subjects took increasingly longer to approach the presented quinine drop in punishment trials but remained faster in comparison to the reward trials (Fig. 4). While increasing their performance in the unrewarded tests in the go/no-go experiment, ants failed to improve beyond chance level. Overall, our results show rapid learning flexibility towards an innate social signal, but also demonstrate a rarely seen hard limit to this flexibility.

Retarded learning due to biological constraints is a well-known phenomenon in animal learning. It appears specifically where certain responses are very difficult or impossible for animals to learn – usually those that lie outside or are in conflict with the animals’ natural responses (Krause, 2015; LoLordo, 1979; Rozin and Kalat, 1971; Seligman and Hager, 1972; Shettleworth, 2009). Rats, for example, can easily form an association between a taste and a subsequent gastric illness (taste aversion) while they fail to associate audio-visual cues with gastric illness or a taste with subsequent electric shocks (Garcia and Koelling, 1966; Wilcoxon et al., 1971). In contrast, vampire bats, a species that only feed on a single kind of food, do not demonstrate taste aversion learning at all (Ratcliffe et al., 2003). However, to date, demonstrations of constraints on learning have only shown a simple inability to apply learning in a particular domain (Domjan, 2005; Krause, 2015; Mineka and Cook, 1988). Here, we provide a very rare demonstration of a case in which a domain is very amenable to learning, but only up to a very well-defined point.

In the present study, we not only investigated the ants’ ability to learn a switch in valence of social signals (compare Bos et al., 2010; Roussel et al., 2012) but also asked our subjects to express the exact opposite of their innate behaviour, making the present experiments, to our knowledge, one of the first assessments of such learning ability in insects. An equivalent in honeybees might be, for example, to attempt to train workers to enter the hive via an unmarked entrance, while avoiding an entrance marked with Nasanov pheromone (Sladen, 1901; von Frisch, 1923).

To conclude, while ants immediately learned to avoid odours when associated with a punishment by assigning negative valence to the stimulus, they failed to do so within 25 trials when the stimulus was a pheromone trail. However, the response to the pheromone was very open to manipulation through experience: ants quickly learned to ignore trails. By developing a simple rule (‘always choose one side and correct if wrong’), they were able to dramatically increase their foraging efficiency. Individual ants can thus develop effective solutions to problems that are beyond their cognitive limitations, by relying on simple rules (Oberhauser et al., 2020).
